# The Importance of Comorbidities at Baseline and 5-Year Follow-Up in a Lung Cancer Biomarker Screening Trial

**DOI:** 10.3390/jcm14062116

**Published:** 2025-03-20

**Authors:** Nimue Lilith Romeikat, Frank Sullivan, Fergus Daly, Wenyan Kong

**Affiliations:** Division of Population & Behavioural Science, Medicine School, University of St. Andrews, St. Andrews KY16 9TF, Scotland, UK; fd46@st-andrews.ac.uk (F.D.); kw247@st-andrews.ac.uk (W.K.)

**Keywords:** national lung cancer screening, lung cancer screening, incidental findings, comorbidity, biomarker, early diagnosis

## Abstract

**Background/Objectives**: Despite recent lung cancer screening (LCS) studies proving significant mortality reduction, comorbidities are a prominent issue affecting cost effectiveness, which is holding back national implementation. Incidental findings (IFs) of comorbidities make a significant contribution to delayed diagnoses and raise discussions about optimal management plans. This is particularly relevant to national lung cancer screening (NLCS), as the high-risk population qualifying for the screening often have increased likelihood for comorbidities due to their smoking history. **Methods**: The Early Detection of Cancer of the Lung Scotland (ECLS) (ClinicalTrials.gov identifier NCT01925625) study showcases a targeted approach to NLCS by implementing the blood-based biomarker EarlyCDT-Lung test. Firstly, this paper explored the ECLS dataset for comorbidities present within the screening population at baseline A chi-square analysis was then undertaken to investigate the relationship of cohort allocation and incidence of new comorbidities over the five-year follow-up period. **Results**: High prevalence conditions were cardiovascular (38.5%), neurological/psychiatric (33.9%), gastrointestinal (29.8%), and respiratory (19.2%). While 20.3% of the total patient cohort showed a newly discovered comorbidity, there was no significant variation in new incidences between the intervention and control cohort. **Conclusions**: When considering these results alongside the all-cause mortality reduction shown in previous analyses, they indicate that this targeted approach to LCS might help improve the benefit–harm ratio through the introduction of biomarkers. Further refining selection criteria for low-dose CT screening might contribute to minimising the risk of overdiagnosis and overtreatment.

## 1. Background

In healthcare systems, where budgets are stretched thin, the need for cost-effective screening has never been more essential. Lung cancer is the most frequently diagnosed cancer and the leading cause of cancer-related deaths worldwide [1]. Despite recent screening studies proving significant mortality reduction, such as the National Lung Screening Trial (NLST) and Dutch Belgian Randomized Lung Cancer Screening Trial (NELSON) [2,3,4,5], a prominent issue regarding cost effectiveness is holding back national implementation in the UK. Incidental findings (IFs) of comorbidities make a significant contribution to this issue, and standardised regulations have not yet been established for the management of all potential IFs. This is especially relevant to lung cancer screening (LCS), as its eligibility criteria includes a smoking history [6]. This leads to a higher prevalence of several comorbidities in the high-risk individuals who qualify for the screening. Kale et al. have modelled the impact of comorbidities on the LCS outcome and showed that the presence of comorbidities at baseline is associated with a reduction in the life years gained by LCS, varying depending on the type of comorbidity [7].

The high prevalence of comorbidities at baseline also manifests in the follow-up of LCS. There has been a wide range of reported IF rates, due to the lack of a standardised reporting system for IFs in low-dose CT (LDCT) LCSs. The NLST reported that 33.8% of participants had a significant IF, of which 89.1% were passed onto the reporting clinician [8]. Most of these IFs include respiratory diseases such as COPD, as well as cardiovascular conditions, namely coronary artery calcifications. In many cases, early detection of comorbidities such as the ones named above may be beneficial; however, dealing with these will entail additional costs as well as benefits. Tsai et al. highlighted that discovery and better management of IFs have the potential to contribute to the all-cause mortality reduction shown with LCSs [9]. A case series study on the NLST population described a large number of IFs [8]. Gareen et al. highlighted that this might pose a significant burden for healthcare systems and have implications for patients due to extensive follow-up testing, but also emphasised that this can be used as an opportunity for “early detection of non-lung cancer in a high-risk population”. Overdiagnosis of conditions which may not affect the patient in their lifetime, or for which there are no effective interventions, may be problematic, however [10]. Tsai et al. also acknowledged that estimating the cost effectiveness of this issue is difficult, as the impact may vary due to variation in healthcare systems and follow-up pathways [9]. A recent study from the US estimated that nearly half (46.2%) of reimbursements by Medicare in the Cleveland Clinic Lung Cancer Screening Program were allocated to the evaluation and treatment of IFs [11]. To implement a cost-effective national LCS programme, a more targeted approach would be beneficial.

There is a lot of discussion on how to determine the scope of lung cancer screening eligibility. Toumazis et al.’s modelling analysis supports the implementation of risk model-based screening strategies, in comparison to the current USPSTF recommendation, which considers age and smoking history to determine screening requirements [12]. Similarly, a meta-analysis and systematic review from February 2025 showed that both the general population and healthcare professionals see personal risk-based screening as a possible alternative to population-based screening [13]. Selection-phase biomarkers are another way to complement current eligibility criteria [14]. This would contribute to reducing overdiagnosis and unnecessary screening in low-risk individuals who would not benefit from undergoing LDCT [15]. As the implementation of a biomarker blood test reduces the number of people receiving a possibly redundant LDCT scan, it may also reduce the burden on healthcare systems associated with the follow-up and management of IFs. It could also facilitate broader adoption by overcoming patient hesitancy toward screening, due to being less invasive and more accessible in comparison to pure LDCT screening models.

Studies such as the Early Detection of Cancer of the Lung Scotland (ECLS) study have implemented an EarlyCDT-Lung test prior to LDCTs, in order to increase early-stage lung cancer diagnosis, whilst mitigating the benefit–risk ratio of LDCTs [2]. The EarlyCDT-Lung test is a blood-based enzyme-linked immunosorbent assay (ELISA) that measures seven autoantibodies—CAGE, GBU4–5, HuD, MAGE A4, NY-ESO-1, SOX2, and p53 [16]. The ECLS study showed that, when using the EarlyCDT-Lung test prior to LDCT, the number of ECLS participants with a late-stage diagnosis of lung cancer was reduced by over a third when compared to standard care. The findings of the five-year analysis were that both lung-specific and all-cause mortality were significantly reduced in patients diagnosed with lung cancer within 2 years after taking the blood-based biomarker test [17]. A different paper has been published comparing ECLS cost effectiveness to the standard of care and LDCT screening for the target population [18].

The purpose of this paper is to explore the comorbidities of participants present at baseline, and whether undergoing biomarker screening is associated with an increased incidence of newly discovered comorbidities within the intervention or control group.

## 2. Materials and Methods

**Figure 1:**
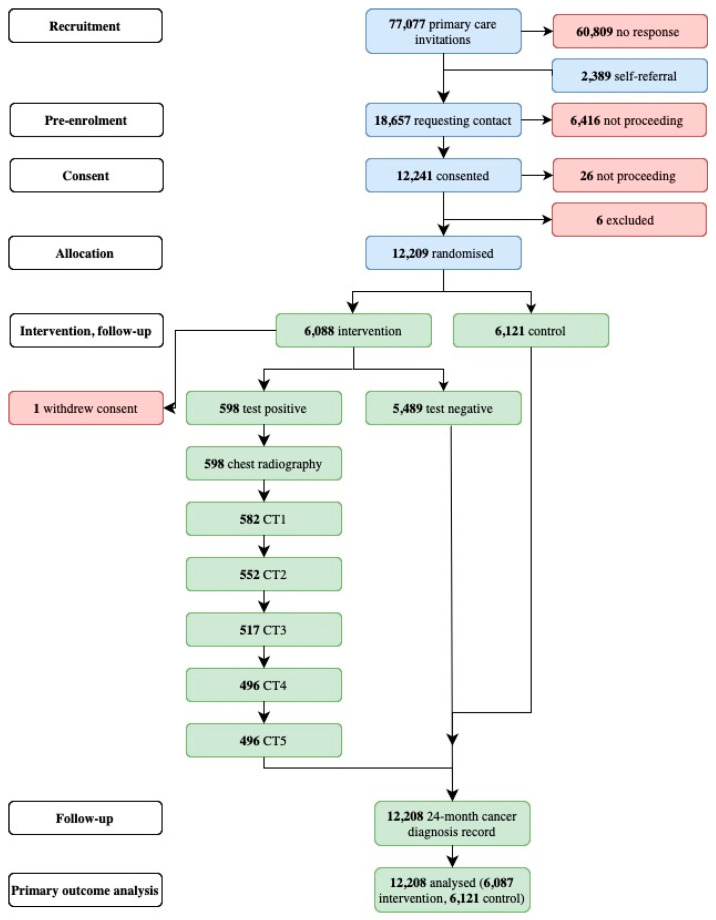
Adapted CONSORT diagram from Sullivan et al., 2017 [19].

We report a secondary analysis on comorbidities in baseline and 5-year follow-up data from the ECLS trial (ClinicalTrials.gov identifier NCT01925625). The ECLS trial was a pragmatic randomised controlled trial, which recruited participants through general practice- and community-based recruitment strategies in Scotland [19], including 12,209 participants (see Figure 1). Each patient allocated to the intervention group undertook an EarlyCDT-Lung test and, if positive, a baseline chest radiograph and 6-monthly chest LDCT scans for 24 months post randomisation. Those in the control group, or those that tested negative in the EarlyCDT-Lung test, received the standardised clinical care provided by NHS Scotland with no further study involvement. Comorbidity data were obtained from the data repository National Services Scotland. The two datasets available were the eDRIS prescription and comorbidity datasets. The eDRIS comorbidity datasets available after the baseline assessment for the ECLS study were filtered for cardiovascular disease, COPD, and malignancy, as these are common comorbidities of individuals with a smoking history. This data limitation was applied to all datasets included within the five-year follow-up analysis to ensure consistency.

The follow-up data are linked to the baseline ECLS datasets, which are stored and managed within the “Safe Haven” in the Trusted Research Environment of the Dundee Health Informatics Centre (HIC). The original ECLS study randomised a total of 12,209 patients, with 6121 allocated to the control group and 6088 to the intervention group, where one withdrew consent. 598 out of the 6088 patients tested positive in the EarlyCDT-Lung test (see Figure 1).

**Figure 2:**
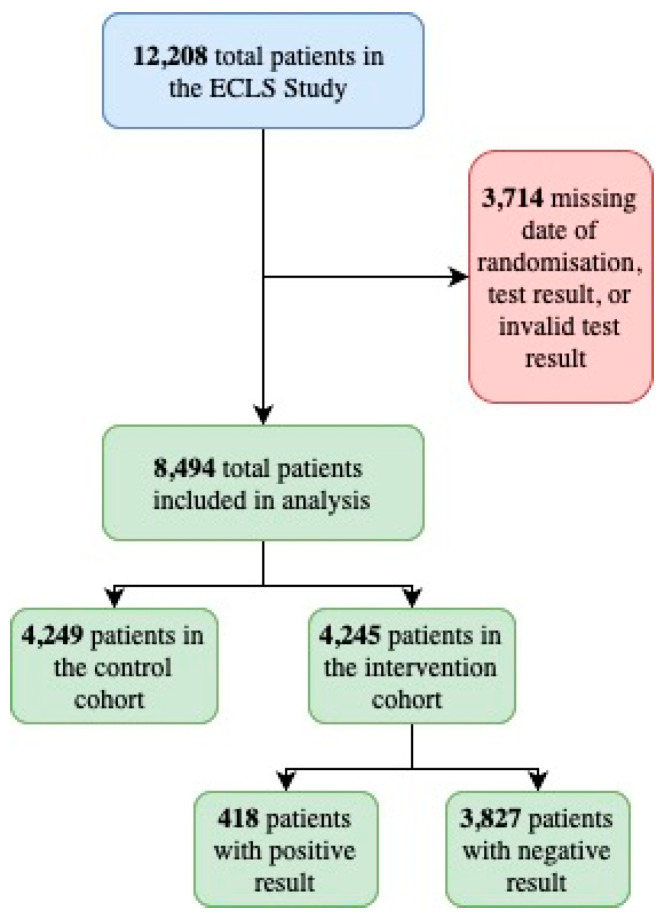
Flowchart of patient inclusion [19].

Patients with a missing date of randomisation, test result, or invalid test result were excluded from this analysis, as this information was relevant for the follow-up analysis. After this exclusion, 8494 patients in total were included in this project. There were 4249 patients in the control and 4245 in the intervention group, of whom 3827 tested negative and 418 tested positive (see Figure 2). Baseline data were established from the timeframe spanning three months prior and following the randomisation date. The 2-year and 5-year follow-up periods were identified as the period between the end of the baseline period and two years or five years after the randomisation date for each patient. An additional 128 patients were excluded for the demographic analysis, as they were missing information on either age, gender, or smoking history.

At randomisation, each patient completed a series of questionnaires exploring their health attitudes, understanding of lung cancer, and past medical history. The questionnaire focusing on the past medical history inquired about mental health conditions, as well as any ongoing healthcare visits, but did not include an extensive record of past comorbidities or a complete list of prescriptions at randomisation. In order to obtain a more complete capture of the range of comorbidities at baseline, eDRIS comorbidity, as well as eDRIS prescription datasets, were utilised. Comorbidities at baseline were thus based upon any repeat prescriptions or healthcare visits within the first three months post-randomisation. OpenPrescribing [20] was utilised to link BNF codes to comorbidity indication. The structures of the BNF codes were taken to group comorbidities into systems: gastrointestinal, respiratory, cardiovascular, neurological/psychiatric, infection, endocrine, genitourinary, malignancy, haematological, musculoskeletal, ophthalmic, ENT, and dermatological. Patients with records of lung cancer (ICD10 codes: C341, C342, C343, C348, and C349) were excluded.

The analytical programme “RScript” was used for all statistical analyses conducted during this study [21]. During the preparation of code for the analysis, Generative AI was utilised for the purpose of contributing ideas to optimise the efficiency of the code. All of these contributions were reviewed by the authors before incorporation into this study. The follow-up analysis had the aim of testing for a relationship between the group allocation and incidence of newly discovered comorbidities during the 5-year follow-up period. Chi-squared testing was used for this univariate analysis to assess the significance of this relationship. As only the patients who tested positive in the EarlyCDT-Lung test were forwarded to LDCT scanning, this analysis was repeated, comparing both testing groups. There were no prescription data available for the five-year follow-up period; thus, this analysis only included ICD10 data for the entire follow-up analysis, in the interest of continuity.

## 3. Results

### 3.1. Summary

This study showed that cardiovascular (38.5%), neurological/psychiatric (33.9%), gastrointestinal (29.8%), and respiratory (19.2%) conditions are highly prevalent within the participants of the ECLS study. The 5-year follow-up analysis showed that there is no significant variation in newly identified comorbidities in the intervention cohort when compared to the control group.

### 3.2. Baseline Analysis

#### 3.2.1. Overview Based on eDRIS Prescription and Comorbidity Data

**Figure 3:**
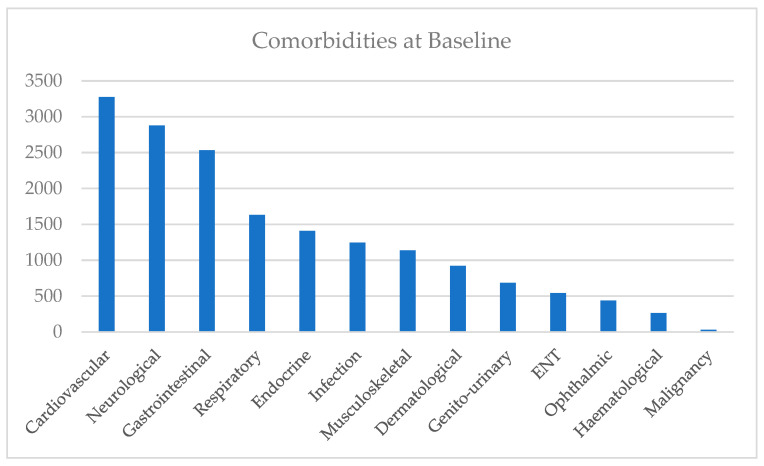
Comorbidities at baseline.

There were 5076 (59.8%) of 8494 total patients with at least one comorbidity at baseline based on eDRIS prescription and ICD10 code data. 4153 (48.9%) of these patients had more than one condition at baseline. The types of comorbidities are shown in Figure 3.

The most common conditions were cardiovascular (38.5%), neurological/psychiatric (33.9%), gastrointestinal (29.8%), and respiratory (19.2%) diseases.

#### 3.2.2. Demographics

There were 8366 patients with full demographic data available, as 128 patients were missing data on either their age, gender, or smoking history. The demographic data show that a higher proportion of participants with comorbidities were older, female, or had a greater number of pack years (see Table 1).

### 3.3. 5-Year Follow-Up

#### 3.3.1. Baseline Overview Based on ICD10 Data

Based on the eDRIS comorbidity ICD10 codes filtered for cardiovascular disease, COPD, or malignancy, there were 129 patients (1.52% of the cohort) with comorbidities at baseline, 62 of whom were in the control cohort, whereas 67 were allocated to the intervention cohort.

#### 3.3.2. Chi-Square Analysis

1722 (20.27%) patients had new incidences of comorbidities within the five years until follow-up. There were 0.25% more newly discovered comorbidities within the intervention cohort (see Table 2).

##### Intervention and Control Cohorts

The chi-square analysis of the incidence of new comorbidities in the intervention cohort compared to the control cohort showed no significant variations between the two (see Table 3). Additionally, the odds ratio shows that the chance of having a new comorbidity discovered is approximately the same in the intervention and control cohorts.

##### Positive Test and Negative Test Cohorts

Similarly, when investigating new comorbidities in the cohort which had positive EarlyCDT-Lung test results compared to those who tested negative, the chi-square analysis did not show any significant variations, as shown in Table 4. This is supported by the odds ratio.

## 4. Discussion

### 4.1. Summary of Findings

The findings of this study show that cardiovascular (38.5%), neurological/psychiatric (33.9%), gastrointestinal (29.8%), and respiratory (19.2%) conditions are frequently observed in the ECLS trial participants at baseline. The 5-year follow-up analysis showed that this targeted screening approach, which utilised a biomarker to select patients prior to LDCT, showed no increase in newly discovered comorbidities in comparison to the control.

Within a smoking population, cardiovascular and respiratory conditions are prevalent comorbidities [22]. This was mirrored in the results of the exploration of comorbidities present at baseline. A cross-sectional study by Barnett et al. stated that, in Scotland, 50–80% of the age group we studied have existing comorbidities [23]. The ECLS study recruited more people in areas of low SE status, where morbidities are outlined to be even more common. Another area that emerged as important was neurological conditions. In this analysis, psychiatric conditions were included within the neurological system. Based on data obtained from the baseline medical history questionnaire, 1418 patients (16.7% of cohort) answered “yes” when asked whether they were taking any prescriptions helping with low mood at the time of the questionnaire. This is in line with the average prescribing rate for antidepressants in Scotland [24]. Gastrointestinal conditions were also shown to be high within this patient population at baseline. Several studies have showcased the detrimental effect that smoking can have on the entire gastrointestinal system [25,26]. The demographic data of the ECLS cohort showed that comorbidities at baseline were more common in females, older participants, and participants with a higher number of pack years.

Based on the five-year follow-up analysis, 20.3% of patients had newly discovered comorbidities. There was, however, no significant difference in these incidences between the intervention and control cohorts, as shown by the chi-square analysis. These results were repeated in the analysis comparing the cohort which tested positive and that which tested negative in the EarlyCDT-Lung test. The findings suggest that there is no significant correlation between the test result and the number of newly identified comorbidities.

The ECLS study differs to other NLCSs in that blood-based testing reduces the number of people needing to undergo imaging. It provides less of an opportunity for IFs, and as a result, fewer comorbidities might be discovered (such as COPD or calcifications indicating cardiovascular conditions). Some studies suggest that IFs such as these can help reduce all-cause mortality in LCS trials [9]. However, the recent 5-year follow-up analysis of the ECLS study showcased that the hazard ratio for all-cause mortality is 0.610 [27]. When considered alongside the reduction in all-cause mortality, the results of this comorbidity analysis imply that this targeted screening approach may help reduce the risk of overdiagnosis and overtreatment resulting from IFs. This contributes to making the ECLS study a cost-effective approach to LCS, overcoming one of the major barriers to national implementation.

### 4.2. Strengths and Limitations

The ECLS study excelled during the community-based recruitment phase by targeting the high-risk population in deprived areas. A study from 2022 reported that these individuals, especially current smokers, tend to be apprehensive to enter screening [28]. Community-based recruitment is incredibly valuable for facilitating effective implementation. Additionally, it provided an objective end-point assessment, applied to both arms of the trial, which enabled a cohort comparison in this analysis. The datasets obtained from the data repository National Services Scotland enabled deterministic data linkage within the HIC and, thus, allowed for high precision in the analyses.

Even though the total number of ECLS participants could not be included in this study, due to individual missing information in the ECLS dataset, this analysis still included a balanced proportion of participants in the control and intervention cohorts. Another point to raise is that the results of the follow-up analysis could have been impacted by the number of multimorbid individuals at baseline. A higher number of multimorbid individuals in one group compared to the other could contort the proportions of newly discovered comorbidities between the two. Based on the ICD10 data, there were eight (0.19% of the cohort) multimorbid patients in the control group and seven (0.16% of the cohort) in the intervention group. Thus, the possibility of multimorbidity posing a limitation to the results is unlikely. Lastly, this paper presents an observational study of routinely collected data. A more specific range of comorbidities at baseline could have been established by undertaking a range of diagnostic routines, such as a glucose tolerance test for diabetes. Equally, the follow-up analysis, investigating the impact of LCS screening on the incidence of new comorbidities being discovered in participants, could have been expanded upon had relevant data been available on the individual follow-up pathways and investigations undertaken by participants after screening.

## 5. Conclusions

Our findings provide a new insight into the baseline comorbidities in a community-based LCS trial in an older and socioeconomically deprived population. The high prevalence of comorbidities at baseline shows the need for the inclusion of a detailed baseline assessment of participants, to avoid undiscovered and severe comorbidities affecting treatment outcome minimising the effectiveness of LCS.

In addition to this, it enhances the comprehension of whether a targeted LCS approach affects the number of newly discovered comorbidities not present at baseline in screened participants. It shows that implementation of a targeted screening approach, like the usage of a biomarker, may minimise the health service demand and patient burden regarding additional follow-up by enhancing selection criteria and only following up with the high-risk population who will benefit from LDCT. Thus, it presents itself as a fitting approach to a national screening programme, where cost effectiveness is a major barrier to implementation.

## Figures and Tables

**Table 1:** Patient Demographics.

Gender	Comorbidity	No Comorbidity	Total	% with Comorbidity
Female	2454	1596	4050	60.6
Male	2539	1777	4316	58.8
Pack Years	Comorbidity	No Comorbidity	Total	% with Comorbidity
0–50	4000	2778	6778	59
51–100	927	554	1481	62.6
101–150	60	37	97	61.9
151–200	3	3	6	50
201–250	3	0	3	100
>250	0	1	1	0
Age	Comorbidity	No Comorbidity	Total	% with Comorbidity
45–55	319	261	580	55
55–65	2972	2108	5080	58.5
66–75	1702	1004	2706	62.9
>75	0	0	0	/

**Table 2:** Incidence of new comorbidities during 5-year follow-up.

Test Group	Malignancy	Cardiovascular Disease	COPD	Total Patients
Control	257	477	426	856
% of cohort	6.05	11.23	10.03	20.15
Intervention	227	516	465	866
% of cohort	5.35	12.16	10.95	20.40
Difference in %	0.70	−0.93	−0.93	−0.25

**Table 3:** Chi-square analysis—intervention and control cohorts.

Comorbidity	*p* Value	Chi-Square Statistic	Odds Ratio	Lower CI	Upper CI
Malignancy	0.163	1.942	0.878	0.730	1.055
Cardiovascular Disease	0.183	1.776	1.094	0.958	1.249
COPD	0.163	1.948	1.104	0.961	1.268
Total Patients	0.770	0.085	1.016	0.914	1.129

**Table 4:** Chi-square analysis—positive test and negative test cohorts.

Comorbidity	*p* Value	Chi-Square Statistic	Odds Ratio	Lower CI	Upper CI
Malignancy	0.590	0.290	0.879	0.549	1.407
Cardiovascular Disease	0.730	0.119	1.055	0.778	1.430
COPD	0.897	0.017	0.979	0.707	1.355
Total Patients	0.871	0.027	0.979	0.761	1.260

## Data Availability

All data were obtained from the data repository National Services Scotland and are stored within the Trusted Research Environment of the Health Informatics Centre Dundee.

## Abbreviations

The following abbreviations are used in this manuscript:

COPD	Chronic obstructive pulmonary disease
ECLS	Early Detection of Cancer of the Lung Scotland study
HIC	Health Informatics Centre
IFs	Incidental Findings
LCS	Lung cancer screening
LDCT	Low-dose CT
NLCS	National lung cancer screening
